# Synthesis and characterization of Magnesium-Iron-Cobalt complex hydrides

**DOI:** 10.1038/s41598-020-65774-8

**Published:** 2020-06-02

**Authors:** Jussara Barale, Stefano Deledda, Erika M. Dematteis, Magnus H. Sørby, Marcello Baricco, Bjørn C. Hauback

**Affiliations:** 1 Department of Chemistry, Inter-departmental Center Nanostructured Interfaces and Surfaces (NIS) and INSTM, University of Turin, Via Pietro Giuria 7, 10125 Torino, Italy; 2 Department for Neutron Materials Characterization, Institute for Energy Technology (IFE), PO Box 40 18, NO-2027 Kjeller, Norway

**Keywords:** Materials for energy and catalysis, Inorganic chemistry

## Abstract

The formation, structure and deuterium desorption properties of Mg_2_Fe_x_Co_(1−x)_D_y_ (0 ≤ x ≤ 1 and 5 ≤ y ≤ 6) complex hydrides were investigated. The synthesis was carried out by reactive ball milling, using a mixture of powders of the parent elements in D_2_ atmosphere. The formation of quaternary deuterides was identified from Rietveld refinements of powder X-Ray diffraction and powder neutron diffraction patterns, and from infrared attenuated total reflectance analysis. It was observed that the crystal structure of deuterides depends on the transition metal fraction. For Co-rich compositions, i.e. up to x = 0.1, hydrides have the tetragonal distorted CaF_2_-type structure (space group *P*4/*nmm*) of Mg_2_CoD_5_ at room temperature. For Fe-rich compositions, i.e. x ≥ 0.5, a cubic hydride is observed, with the same K_2_PtCl_6_-type structure (space group *Fm*$\bar{{\bf{3}}}$*m*) as Mg_2_FeD_6_ and as Mg_2_CoD_5_ at high temperatures. For x = 0.3, both the cubic and the tetragonal deuterides are detected. Differential scanning calorimetry coupled with thermogravimetric and temperature programmed desorption analyses show rather similar deuterium desorption properties for all samples, without significant changes as a function of composition. Finally, hydrogen sorption experiments performed for Mg_2_Fe_0.5_Co_0.5_H_5.5_ at 30 bar of H_2_ and 673 K showed reversible reactions, with good kinetic for both absorption and desorption of hydrogen.

## Introduction

Hydrogen is a promising energy carrier, but technologies for its storage must be improved for its use in a large scale. The purpose of research on hydrogen storage is to achieve high gravimetric and volumetric capacities at mild pressure and temperature conditions. In this regard, hydrogen storage in hydrides is particularly favourable^1^. Mg is an attractive hydrogen storage material, due to its low cost and high gravimetric (7.7 H_2_ wt.%) and volumetric (110 gH_2_l^−1^) capacity in MgH_2_. However, MgH_2_ is a rather stable hydride and its desorption temperature is too high (>573 K) for most practical applications. To reduce the desorption temperature and increase the kinetics of both absorption and desorption, different strategies have been investigated, such as the introduction of defects, the reduction of particles size (e.g. with mechanochemical techniques) and the use of additives (e.g. 3d transition metals and their oxides)^2^. Mg-based hydrides containing 3d transition metals (TM), e.g. Ni, Fe and Co, have shown lower hydrogen sorption temperature and improved kinetics, compared to MgH_2_.

Mg_2_FeH_6_ and Mg_2_CoH_5_ have high gravimetric (5.6 and 4.5 H_2_ wt.%, respectively)^3^ and volumetric (150 and 110 gH_2_l^−1^, respectively) capacity^4^. The crystal structure of both these ternary hydrides is based on the formation of complex anions obeying the 18-electron rule and with a strong covalent bond between TM and hydrogen. In Mg_2_FeH_6_, the octahedral complex anion [FeH_6_]^4−^ is surrounded by eight Mg^2+^ in a cubic rearrangement. The crystal structure is a cubic K_2_PtCl_6_-type (space group *Fm*$\bar{3}$*m*), with unit cell parameter *a* = 6.430(1) Å^3^. Measurements by infrared spectroscopy indicates a single vibration band at 1729 cm^−1^, that is shifted to 1262 cm^−1^ if deuterium substitutes hydrogen^3^. Mg_2_CoH_5_ has a tetragonally distorted CaF_2_-type structure at room temperature (RT), with space group *P*4/*nmm* and lattice parameters *a* and *c* of 4.483(2) Å and 6.599(6) Å, respectively^5^. The [CoH_5_]^4−^ complex anion has a square pyramidal arrangement and it is surrounded by four Mg^2+^ cations. The two different Co-H bonds in the structure have different lengths. From neutron diffraction measurements, using deuterium instead of hydrogen, the Co-D distance at the basis of the pyramid has been observed at 1.515(3) Å, while the apical deuterium is at a distance of 1.590(17) Å^3^. Thus, [CoH_5_]^4−^ displays two infrared stretching bands at 1757 and 1632 cm^−1^, which are shifted to 1275 and 1173 cm^−1^ with deuterium^6^. At 488 K the hydride undergoes an allotropic transformation, resulting in the same cubic structure as Mg_2_FeH_6_^7^.

Fe is fully immiscible in Mg^8^, while Co forms a stable intermetallic compounds (MgCo_2_)^9^. Thus, neither of the TMs form stable intermetallic compounds Mg_2_TM to be used as precursor for the hydride^10^. This means that the synthesis of ternary complex hydride is challenging, since the direct hydrogenation of the intermetallic phase is not possible. Thus, it is necessary to proceed with a reaction between the TMs and MgH_2_ or Mg under hydrogen atmosphere, as summarized by the two reactions below:

1 $$2{{\rm{MgH}}}_{2}+{\rm{TM}}+(x-2){{\rm{H}}}_{2}\to {{\rm{Mg}}}_{2}{{\rm{TMH}}}_{2x}$$

2 $$2\,{\rm{Mg}}+{\rm{TM}}+{x}\,{{\rm{H}}}_{2}\to {{\rm{Mg}}}_{2}{{\rm{TMH}}}_{2x}$$

It should be noted that, Eq. 2 can occur via the intermediate formation of MgH_2_, after which the reaction proceeds according to Eq. 1^11^. In general, the route governing the formation of Mg_2_TMH_*x*_ complexes strongly depends on the synthesis method and processing conditions^10,12–17^. Regardless the route, it has been proposed that hydrogen is attracted to Mg(H_2_)/TM interfaces in order to reduce the interfacial energy, which is positive due to both topological disorder and the positive heat of mixing of Mg and Fe or Co^4^.

A conventional method of synthesis, such as annealing, requires a reaction at high temperature (e.g. >673 K) and high hydrogen pressure (e.g. 50-100 bar) for relatively long reaction times (i.e. more than one day), and only provides yields around 50%^18,19^. On the other hand, mechanochemical synthesis methods in a reactive atmosphere allow for the formation of those hydrides at lower hydrogen pressures (i.e. ≤50  bar) and close to RT, reaching yields of more than 80%^20,21^. The reactive mechanochemical synthesis has the advantage of enhancing hydrogen sorption kinetics, due to the formation of fresh surfaces and reduced particle sizes^22^, allowing hydride formation already after few hours of milling^21,22^.

In the last few years, some interest has been devoted to Mg-based complex hydrides with more than one TM and the formation of quaternary hydrides with Mg, Fe and Co has been already reported^4,11,23^. Baum *et al*.^11^ presented the formation of Mg_2_(FeH_6_)_0.5_(CoH_5_)_0.5_, hinting at a complex formation process, due to the immiscibility of Fe and Co with Mg. Deledda and Hauback^4^ reported in more details the structure and thermal stability of Mg_2_(FeH_6_)_0.5_(CoH_5_)_0.5_, showing that the quaternary hydride is isostructural to Mg_2_FeH_6_ and to the high-temperature phase of Mg_2_CoH_5_, containing both [FeH_6_]^4−^ and [CoH_5_]^4−^ complex anions^4^. Moreover, they showed a hydrogen desorption temperature of 570 K, intermediate to 560 K for Mg_2_FeH_6_ and 585 K for Mg_2_CoH_5_. Finally, Zélis *et al*.^23^ investigated the synthesis of mixed Mg_2_(FeH_6_)_(1−x)_(CoH_5_)_x_ systems, with different Fe-Co contents (x = 0.25, 0.5, 0.75). However, no detailed structural and thermal characterizations were reported, suggesting that further studies on this system are necessary.

The aim of this work is to synthetize and investigate the properties of Mg_2_Fe_x_Co_(1−x)_D_y_ complex deuterides with different Fe-Co and D contents (0 ≤ x ≤ 1 and 5 ≤ y ≤ 6), comparing results with the ternary Mg_2_CoD_5_ and Mg_2_FeD_6_ compounds. Deuterium was used instead of hydrogen to allow the structural study with Powder Neutron Diffraction (PND). The use of neutron diffraction is crucial for characterizing the crystalline structure, since it allows to distinguish Fe and Co (which have very similar X-ray scattering cross section) and to determine the occupancy and the position of deuterium (i.e. H). The quaternary deuterides/hydrides synthesized in this study where found to be isostructural either with Mg_2_CoD_5_ (tetragonal *P*4/*nmm*), for x = 0.1, or with Mg_2_FeD_6_ (cubic *Fm*$\bar{3}$*m*) for x ≥ 0.46. For x = 0.3, two hydrides are formed: Mg_2_(FeD_6_)_0.3_(CoD_5_)_0.7_ (tetragonal *P*4/*nmm*) and Mg_2_(FeD_6_)_0.4_(CoD_5_)_0.6_ (cubic *Fm*$\bar{3}$*m*). All hydrides have a similar hydrogen desorption process, with a maximum desorption temperature T_max_ ≅ 550 K and activation energy of desorption Ea_des_ ≅ 95 kJmol^−1^. The enthalpy of desorption has been used to determine the thermodynamics of tetragonal and cubic solid solutions. Rehydrogenation of Mg_2_(FeH_6_)_0.5_(CoH_5_)_0.5_ occurs at 673 K in 30 bar of hydrogen with relatively good kinetics.

## Experimental

### Synthesis

The synthesis of quaternary deuterides, with formula Mg_2_Fe_x_Co_(1−x)_D_y_ was achieved by Reactive Ball Milling (RBM) using a deuterium atmosphere inside the milling vial. The nominal amount of iron and cobalt is given by x (x = 0.1, 0.3, 0.5, 0.7 and 0.9) and refers to the nominal content of Fe and Co in the starting elemental powder mixtures. For simplicity, we refer to those samples as Fe0.1, Fe0.3, Fe0.5, Fe0.7, Fe0.9, respectively. The ternary compounds Mg_2_CoD_5_ and Mg_2_FeD_6_, referred as sample Fe0.0 and Fe1.0, respectively, were also prepared for comparison.

The synthesis was carried out using elemental powder of Fe (200 mesh), Co (350 mesh) and Mg (350 mesh) with a purity level over 99% (purchased from Alfa Aesar) and deuterium gas (purchased from Nippon Gases) with purity >99.5%. No further gas purification process was applied. The milling was carried out in a Fritsch Pulverisette 6 (P6) planetary ball milling, using a specially designed hardened steel vial, commercialized by Evico Magnetics. The vial is rated to 150 bar and equipped with a temperature and pressure monitoring system. In this work, milling was carried out in 50 bar of D_2_, at RT, for 20 hours at 400 rpm, using 10 mm diameter hardened steel balls, with a ball-to-powder weight ratio of approximately 40:1. The amount of deuterium absorbed during milling was calculated applying the perfect gas law from the changes in pressure and temperature recorded by the monitoring system and by taking into account the free volume within the vial. After the synthesis, about 200 mg of the as-milled powders were annealed to reduce the internal stresses created by milling, allowing an accurate structural characterization. The thermal treatment was performed at 473 K in 50 bar of D_2_ for a period of approximately 48 hours. All samples were handled in a glovebox in a purified Ar atmosphere.

### Structural characterization

#### Powder X-ray Diffraction

Powder X-Ray Diffraction (PXD) analysis was performed using a Bruker D8 A25 diffractometer. It was equipped with Mo K-α radiation and a Lynxeye detector. The powder samples were packed in glass capillaries with a diameter of 0.5 mm. The PXD scan speed was 2 s per step, with steps of 0.04° from 5° to 45° in 2θ. Rietveld refinements were carried out with the software Maud^24^ and Topas v6.0^25^. PXD was also performed using a Gemini R-Ultra diffractometer, to study the rehydrogenated sample. The instrument operates in Debye-Scherrer geometry and it is equipped with a Mo K-α source and a CCD detector. The samples were mixed with a paraffin oil in glovebox and fixed on the tip of a needle, which rotates during acquisition.

#### Powder neutron diffraction

Annealed samples were analysed by PND with the high-resolution neutron diffractometer PUS^26^ at the JEEP II research reactor in Kjeller (Norway). The beam had a wavelength, λ = 1.5546 Å. The samples were sealed in cylindrical vanadium holders with 6 mm diameter. The diffraction patterns were acquired at RT from 10° to 130 ° in 2θ by two detector banks with 7 position-sensitive ^3^He-filled detector tubes in each. In this case, Rietveld refinement was carried out also with FullProf software^27^.

#### Attenuated total reflectance

Attenuated Total Reflectance Infra-Red spectroscopy (ATR FT-IR) was carried out in a glovebox, using an ALPHA FT-IR spectrometer from Bruker. The measurements were performed using a Ge crystal as reflection element. The spectra were obtained in the range of 4000-400 cm^−1^ with a resolution of 2 cm^−1^.

#### Scanning Electron Microscopy

Powders were analyzed by Scanning Electron Microscopy (SEM), using a Zeiss EVO 50 XVP-LaB_6_ equipped with an Oxford Instrument INCA Energy 250 for EDS analysis. Measurements were performed at 20 kV and 100 mA, using backscattered electrons.

### Thermal characterization

#### Differential Scanning Calorimetric coupled with Thermal Gravimetric Analysis

The thermal stability was studied by Differential Scanning Calorimetry coupled with Thermal Gravimetric Analysis (DSC-TGA). Measurements were performed on a STA 449 F3 Jupiter instrument produced by Netzsch, and carried out with an Argon flow of 50 ml/min. The as-milled powders were heated at different heating rates (1, 5, 10, 20 and 40 K/min), from RT up to a maximum of 873 K. The maximum peak temperatures T_max_ of the endothermic events obtained at different heating rates were used to calculate the desorption activation energy (Ea_des_) using the Kissinger method^28^, after having verified the isokinetic conditions^28^. In addition, the enthalpy of hydrogen desorption (ΔH_des_) was estimated by integrating the area of the endothermic peaks in the DSC trace.

#### Thermal programmed desorption

Thermal Programmed Desorption (TPD) was performed using an instrument built in-house, which is equipped with a diaphragm pump and a turbomolecular pump to reach 10^−5^ mbar. As-milled powders were heated at 5 K/min from RT up to 823 K in vacuum.

### Volumetric measurements by Sievert’s Method

Hydrogen sorption measurements were performed by Sievert’s method with a volumetric apparatus from AMC (Pittsburgh). Analysis were performed with hydrogen gas (purchased from Nippon Gases) with purity >99.9999%. Measurements were carried out in isothermal conditions at 673 K. Desorption was obtained in vacuum for a maximum of 10 h, using a rotary pump (10^−2^ bar), while absorption was performed at 10 bar and 30 bar of H_2_ for a maximum of 35 h.

## Results and discussion

### Synthesis with reactive ball milling

**Figure 1:**
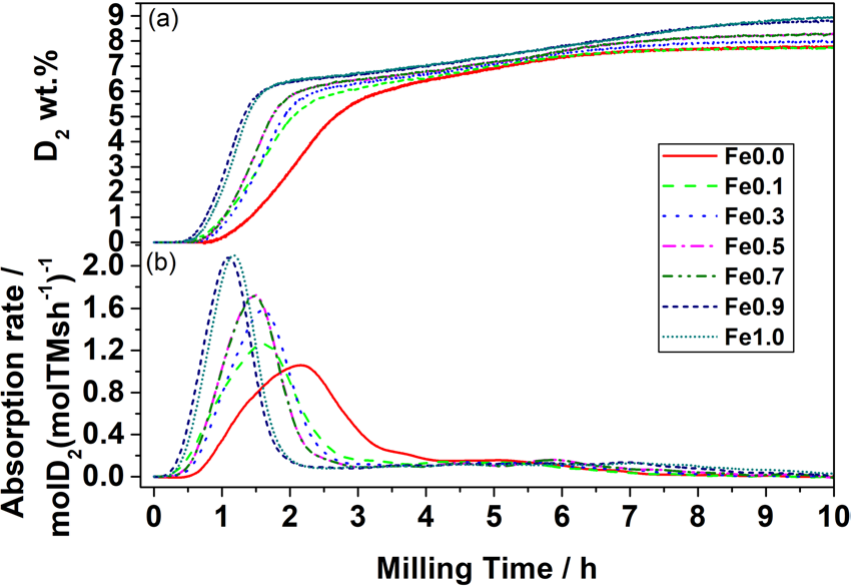
(**a**) Gravimetric deuterium capacity expressed as D_2_ wt.% and (**b**) absorption rate as a function of milling time.

Figure 1a shows D_2_ absorbed by the powders as a function of milling time. After an incubation time ranging from 30 to 50 min, absorption starts and continues for about 8-10 h. A high rate of hydrogenation is observed during the first 2 hours, followed by a slower absorption up to the end of the hydrogenation. The latter can be linked to the progressive consumption of fresh Mg/Fe-Co interfaces, which results in slower hydrogenation rates towards the end of the milling process^4^. This trend can be better observed by plotting the absorption rate as a function of milling time (Fig. 1b). The higher the amount of iron, the higher the rate of hydrogenation. Even a small amount of Fe drastically increases the hydrogen absorption kinetics, as seen by comparing Fe0.0 (no Fe) and Fe0.1 (nominal amount of Fe 0.1) in Fig. 1b.

**Figure 2:**
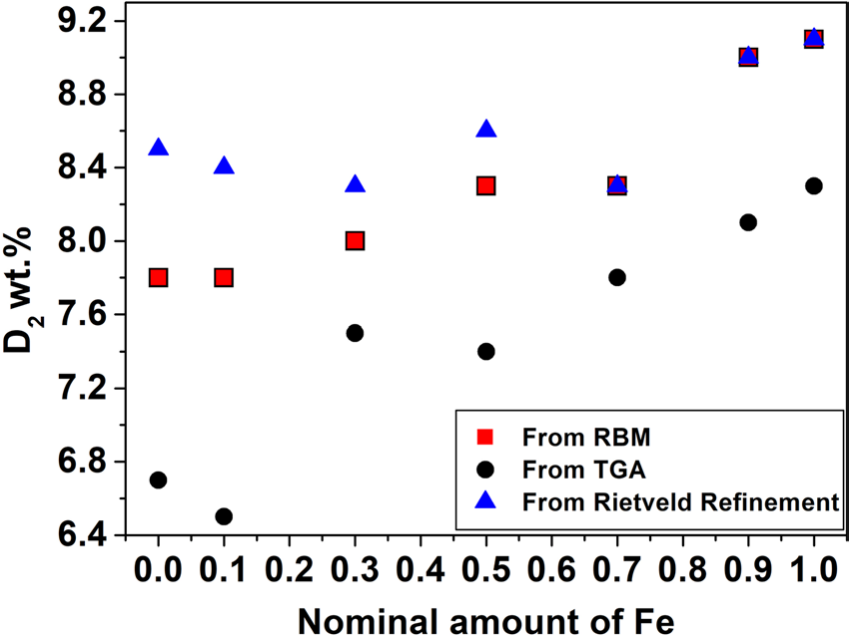
Gravimetric deuterium capacity calculated from the pressure changes recorded during Reactive Ball Milling (red squares), from TGA (black dots) and from Rietveld refinements (blue triangles) as a function of the nominal amount of iron.

The deuterium content obtained after RBM is reported as a function of the nominal amount of iron in Fig. 2. A rather linear trend is observed, reflecting the ability of Fe to coordinate more deuterium atoms (six) with respect to Co (five). On the other hand, there are no significant differences between the two ternary hydrides and Fe or Co rich ones (i.e. Fe0.0-Fe0.1 and Fe1.0-Fe0.9), respectively. Samples Fe0.3, Fe0.5 and Fe0.7 also absorb a comparable amount of deuterium.

### Structural characterization of as-milled and annealed samples

PXD patterns of all samples annealed at 473 K in 50 bar of D_2_ are shown in Fig. S1. In sample Fe1.0, some unreacted iron is detected, while in sample Fe0.0 no excess of elemental Co is observed. In all other mixtures, the presence of a bcc-(Fe,Co) phase is observed, suggesting a non-complete hydrogenation. PND patterns are shown in Fig. S2. The results of Rietveld refinements with the PXD patterns were used as starting point for further refinements with the PND data.

The refinements with the PND patterns show the formation of the tetragonal Mg_2_CoD_5_ phase in Fe0.0 and the cubic Mg_2_FeD_6_ in Fe1.0, respectively. Tetragonal and cubic phases are observed for all other intermediate compositions, with Fe and Co randomly occupying the same site in the structure, as reported by Deledda and Hauback for Mg_2_(FeD_6_)_0.5_(CoD_5_)_0.5_^4^.

**Figure 3:**
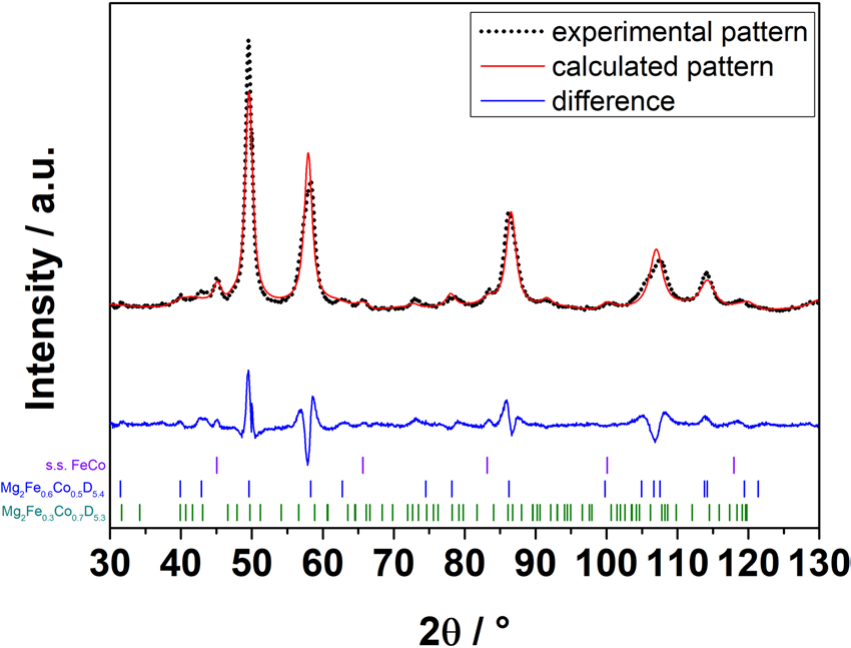
Refined (red line) and experimental (black dots) PND patterns of sample Fe0.3. The difference between observed and calculated intensities is also shown (blue line), together with the position of Bragg reflections for FeCo, Mg_2_Fe_0.4_Co_0.6_D_5.4_ and Mg_2_Fe_0.3_Co_0.7_D_5.3_.

Table 1 summarizes the structural information obtained from the Rietveld refinements, including the formula obtained from the refinements of the occupancy. From Table 1, we can see that adding Fe in the tetragonal structure, is causing a decrease in the unit cell parameter *c*, while *a* is increasing. The addition of Co in the cubic structure of Mg_2_FeD_6_ causes a decrease in unit cell dimension. Figures 3 and S3 show the experimental and calculated PND patterns for sample Fe0.3 and Fe0.7, respectively.

**Table 1:** Sample names, compositions, and structural information obtained by Rietveld refinement of PND data for all the samples synthesized in this work.

Sample	Hydride Formula	Space Group	Cell parameters [Å]		V cell [Å^3^]	Yeld [wt.%]	Ref. cell parameters [Å]	
Fe0.0	Mg_2_CoD_5_	*P*4/*nmm*	*a* 4.474(8)	*c* 6.559(7)	131.3(5)	100	*a* 4.483(2)	*c* 6.599(6)^5^
Fe0.1	Mg_2_(FeD_5_)_0.09_(CoD_5_)_0.91_	*P*4/*nmm*	*a* 4.485(5)	*c* 6.529(1)	131.37(6)	97	*—*	
Fe0.3	Mg_2_(FeD_6_)_0.30_(CoD_5_)_0.70_	*P*4/*nmm*	*a* 4.494(1)	*c* 6.523(2)	264.6(1)	57	—	
	Mg_2_(FeD_6_)_0.40_(CoD_5_)_0.60_	*Fm*$\bar{3}$*m*	6.419(1)		264.6(1)	34		
Fe0.5	Mg_2_(FeD_6_)_0.50_(CoD_5_)_0.50_	*Fm*$\bar{3}$*m*	6.420(5)		264.6(7)	91	*a* 6.426^4^	
Fe0.7	Mg_2_(FeD_6_)_0.70_(CoD_5_)_0.30_	*Fm*$\bar{3}$*m*	6.428(1)		265.6(1)	85	—	
Fe0.9	Mg_2_(FeD_6_)_0.84_(CoD_6_) _0.16_	*Fm*$\bar{3}$*m*	6.423(6)		265.0(5)	89	—	
Fe1.0	Mg_2_FeD_6_	*Fm*$\bar{3}$*m*	6.430(4)		265.8(9)	88	*a* 6.430(1)^3^	

The yield calculated from Rietveld refinement of PXD data is also reported together with the cell parameters reported in literature.

The refinements confirm the formation of a cubic phase in Fe09, Fe0.7 and Fe0.5. For the latter, results agree with ref. ^4^. The hydrogen volumetric capacity calculated from the unit cell volume is 138 g/l for Mg_2_Fe_0.5_Co_0.5_D_5.5_ and 143 g/l for Mg_2_Fe_0.7_Co_0.3_D_5.7_, suggesting similar hydrogen storage capacities.

For sample Fe0.3 the structural analyses are more challenging. The peaks at 60° and 110° in 2θ (Fig. 3), hints at the presence of a structure which is not purely cubic. The peak at 60° shows a shoulder, which might suggest the influence from a tetragonal phase. Indeed, refinements with a single cubic phase resulted in a poor fit. On the other hand, refinements with a single tetragonal phase with Mg_2_CoH_5_-type structure did not yield a satisfactory fit either. Therefore, a refinement was performed considering the presence of the two pure ternary hydrides, Mg_2_CoD_5_ and Mg_2_FeD_6_, but it was impossible to reach convergence and the unit cell parameters were far from the expected values. However, refinements with both a cubic phase with Co substituting Fe and a tetragonal phase with Fe substituting Co, together with a ~9 wt% of unreacted metallic Fe, yield excellent fit to both the PND and PXD data. To decrease the number of refinable parameters, the composition of the cubic phase was fixed to Mg_2_(Co_0.6_Fe_0.4_)D_5.4_ as estimated from the unit cell parameter (*a* = 6.419(1) Å). The Fe/Co ratio in the tetragonal phase was refined with a soft constraint on keeping the overall elemental composition of the model phases similar to the nominal composition. The deuterium coordination around the transition metal site was set to a fully occupied square pyramid, similar to that in Mg_2_CoD_5_, with an additional, partly occupied apical site below the basal plane. The occupancy of the addition apical site was set to be equal to the occupancy of Fe on the transition metal site, thus accounting for the expected octahedral D coordination around the Fe atoms. The difference between refined and nominal elemental compositions was less than 7 wt% for all elements. A model where both the apical sites in the tetrahedral phase were partly occupied was also tested but yielded slightly poorer fits.

Zélis *et al*.^23^ previously reported on the quaternary hydride Mg-Fe-Co-H with a Fe:Co ratio of 0.25:0.75. Based on PXD analysis, they assigned a tetragonal symmetry *P*4/*nmm*, isostructural with Mg_2_CoH_5_. Moreover, using Mössbauer spectroscopy, a non-cubic symmetry was revealed for the Fe sites^23^. Thus, considering the results reported by Zelis *et al*.^23^ and the one obtained in this work, we can conclude that for 0 ≤ x ≤ 0.25 there exists a tetragonal hydride Mg_2_Fe_x_Co_(1−x)_D_y_ with 5 ≤ y ≤ 5.25. Around x = 0.3, a two-phase region is found where the tetragonal hydride Mg_2_Fe_x_Co_(1−x)_D_y_ coexists with a cubic hydride, as that formed for 0.5 ≤ x ≤ 0.9. It is worth noting that these results agree with Verbovytskyy *et al*.^29^ who found a tetragonal *P*4/*nmm* symmetry, isostructural with Mg_2_CoH_5_, for the quaternary hydride Mg_2_Ni_0.5_Co_0.5_H_4.4_ and suggested that quaternary Mg_2_M_x_M’_y_H_x_ hydrides display the same the structure of the parent ternary phases.

**Figure 4:**
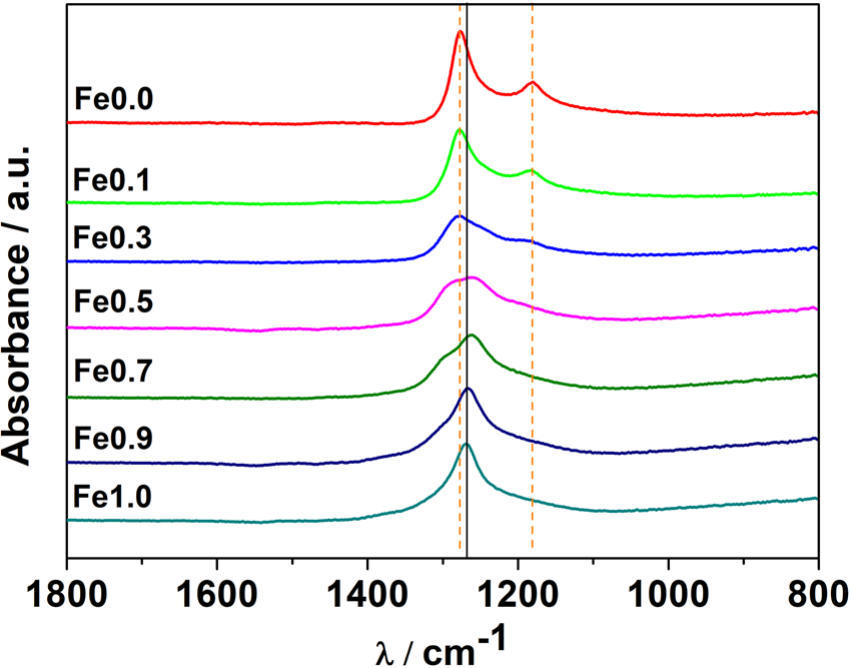
FT-IR spectra for all samples. Orange dashed lines indicate [CoD_5_]^4−^ bands (1275 and 1173 cm^−1 ^^6^), while the black full line refers to [FeD_6_]^4−^ (1262 cm^−1 ^^3^).

For all samples, ATR-IR spectra are shown in Fig. 4. As reported in refs. ^3,6^, [FeD_6_]^4−^ shows one stretching band, while [CoD_5_]^4−^ shows two bands, which reflect the octahedral and square-base pyramidal symmetry, respectively. They appear in the same frequency range. The observed frequencies are in good agreement with literature^3,6^: for sample Fe1.0, the [FeD_6_]^4−^ stretching is observed at 1261 cm^−1^, while, for Fe0.0, the two bands for [CoD_5_]^4−^ are around 1180-1200 cm^−1^ and 1278-1290 cm^−1^. The formation of the quaternary hydride implies the presence of both complex anions in the structure and this can be clearly observed in Fe0.3, Fe0.5 and Fe0.7. For example, in Fe0.7, the higher amount of Fe with respect to Co, implies a main contribution of [FeD_6_]^4−^, so that the observed band is similar to that of Fe1.0. The presence of Co in the structure results in a broader band with respect to that of Fe1.0, with shoulders due to the [CoD_5_]^4−^ bands. On the other hand, ATR-IR spectra of Fe0.1 and Fe0.9, do not clearly show the presence of both complexes (Fig. 4). This is likely due to the sensitivity limits of the instrument, since the presence of [FeD_6_]^4−^ in Fe0.1 and [CoD_5_]^4−^ in Fe0.9 was confirmed by PND and PXD.

Table 1 shows also the amount of hydride phase formed, obtained from the refinements with PXD patterns, using crystal structure information established by PND. The amount of hydride formed after milling is in all cases higher than 80%. Such high yields have been obtained thanks to the use of high-energy milling techniques, confirming that it is more suitable for the synthesis of Mg-Fe-Co hydrides with respect to conventional sintering methods^18,19^. Using the quantitative results, a mass balance was performed to calculate the amount of D_2_ absorbed in all samples (Fig. 2). In most cases, the results confirm the amount obtained by RBM reported in Fig. 2, except for the Fe0.0 and Fe0.1, which have an error of 9 and 8%, respectively.

It can be concluded that the quaternary hydride structure is influenced by the amount of complex anions present. In all five mixtures investigated in this study, Fe and Co randomly occupy the same site, creating solid solutions with a tetragonal structure of Mg_2_CoD_5_ for Co-rich samples and with a cubic structure of Mg_2_FeD_6_ for Fe-rich ones. The hydride formed are Mg_2_(FeD_6_)_0.09_(CoD_5_)_0.91,_ Mg_2_(FeD_6_)_0.3_(CoD_5_)_0.7_, Mg_2_(FeD_6_)_0.4_(CoD_5_)_0.6_, Mg_2_(FeD_6_)_0.5_(CoD_5_)_0.5_, Mg_2_(FeD_6_)_0.7_(CoD_5_)_0.3_ and Mg_2_(FeD_6_)_0.9_(CoD_5_)_0.1_. Finally, a two-phase region was found around x = 0.3.

### Thermal stability

**Figure 5:**
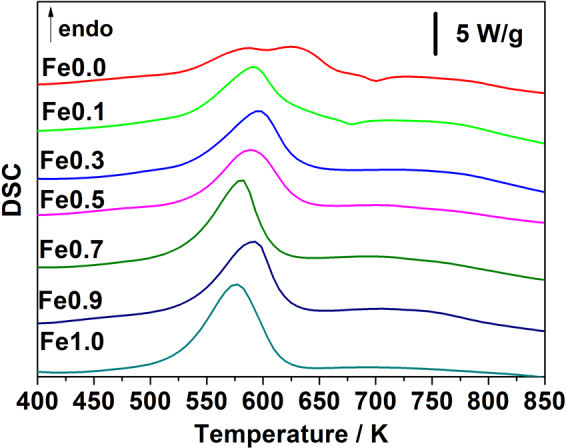
DSC traces recorded at 20 K/min for all as-milled samples and plotted as a function of temperature.

Results of the DSC analysis at 20 K/min for all samples are shown in Fig. 5. The decomposition of the hydride phase occurs in a single step, except for Fe0.0 and Fe0.1. Indeed, at high heating rates (20 K/min in Fig. 5 and 40 K/min in Fig. S4), the desorption clearly presents two overlapping peaks, while in the analysis at lower heating rates (1, 5, 10 K/min in Figs. S5–S7) only a broad desorption peak is detected. This agrees with previous reports^5,7,17^ which claim that hydrogen desorption of Mg_2_CoH_5_ can involve the formation and successive decomposition of other Mg-Co hydrides (i.e. Mg_6_Co_2_H_11_^30^ or Mg_3_CoH_5_^5^). However, in Fe0.1 the double peak is less pronounced, suggesting that small amounts of Fe can affect the desorption mechanism of the tetragonal Mg_2_CoD_5_ phase. This is not observed for Fe0.3. In this sample, two hydrides need to desorb (section 3.2), but two separate signals are not detected. This indicates similar desorption temperatures of the two hydrides. In the samples Fe0.0 and Fe0.1, a small exothermic peak is also observed at about 650-700 K and is associated to the formation of the intermetallic compound MgCo^5,17^. This is a metastable phase, as presented in the introduction, only MgCo_2_ is stable in the Mg-Co system^9^.

From the comparison of the derivative of TG curves with DSC and TPD traces at 5 K/min (Figs. S8, S6 and S9, respectively), it can be clearly observed that the weight loss is associated with a deuterium desorption event. As seen from the DSC signals (Figs. 5 and S4–S7) and Table 2, summarizing the values of the maximum temperature of the desorption peaks registered from TPD analysis at 5 K/min, the ternary and quaternary hydrides have similar temperatures of desorption. This is in contrast with TPD analysis at 2 K/min in ref. ^4^, in which it was found that Mg_2_FeH_6_ has a maximum of desorption temperature at 560 K, Mg_2_CoH_5_ at 585 K (a single desorption peak is reported) and Mg_2_(FeH_6_)_0.5_(CoH_5_)_0.5_ at 570 K. This discrepancy can be related to the mechanism governing hydrogen desorption in Mg_2_CoH_5_, which is not yet fully explained. The occurrence of a double peak observed here has been also reported earlier, even for low heating rates^5,30^ and is related to the formation and decomposition of Mg_6_Co_2_H_11_^30^ or Mg_3_CoH_5_^5^. Zépon *et al*.^31^ proposed two different mechanisms, depending on temperature, for hydrogen desorption. At low temperatures, hydrogen is released without any structural changes of the hydride. However, at temperatures above 573 K, another mechanism involving the formation of Mg_2_CoH_x<5_, which then decomposes up to the end, takes place^31^. The discrepancy between this work and ref. ^4^. can therefore be linked to the complexity of the Mg_2_CoH_5_ decomposition. Thus, we cannot confirm that the thermal behaviour of the quaternary hydrides is intermediate to that of ternary hydrides^4^, but it can be concluded that it is rather similar.

**Table 2:** Summary of the values of the activation energy for hydrogen desorption (Ea_des_) obtained by Kissinger method from DSC and TGA data together with the values of enthalpy of desorption (ΔH_des_) measured from the DSC peaks. The values of Ea_des_ and ΔH_des_ obtained from the literature are also reported for comparison.

Sample	Ea_des_ from DSC [kJmol^−1^]	Ea_des_ from TGA [kJmol^−1^]	Ea_des_ Ref. [kJmol^−1^]	ΔH_des_ from DSC [kJmol^−1^D_2_]	ΔH_des_ Ref. [kJmol^−1^D_2_]	T_max_ from TPD [K]
Fe0.0	76	104	114.8^5^	56 ± 8	54^32^	529
Fe0.1	91	107	—	69 ± 12	—	538
Fe0.3	89	105	—	69 ± 5	—	543
Fe0.5	99	106	—	70 ± 9	—	537
Fe0.7	95	100	—	74 ± 7	—	538
Fe0.9	89	94	—	74 ± 10	—	543
Fe1.0	87	81	90–100^13^	73 ± 7	77.4^33^	530

Finally, the maximum TPD peak temperatures (T_max_) recorded at 5 K/min are also listed.

Figure 2 reports the D_2_ weight loss registered from TG analysis at 5 K/min. The results are slightly lower than the amount absorbed during the synthesis. This underestimation is likely caused by a slight sample oxidation during the TG measurements, since the instrument is not placed in a glovebox and samples are air sensitive. Table 2 presents the experimental values of ΔH_des_ obtained from the DSC analysis and the Ea_des_ calculated applying the Kissinger method (see section 2.3.1.) on DSC traces and on the derivative of the TG curves (Fig. S10). As can be seen from Table 2, the Ea_des_ data obtained from the two techniques are similar, except for Fe0.0. For the ternary hydrides, ΔH_des_ and Ea_des_ are in good agreement with literature values^14,32,33^ (Table 2**)**, except for Ea_des_ of Mg_2_CoH_5,_ which is significantly lower than 114.8 kJ/mol reported by Norek *et al*.^5^. Results for the hydrides for samples from Fe0.1 to Fe 0.9 are comparable, with values of Ea_des_ 89-99 kJmol^−1^ and ΔH_des_ 69-74 kJ/mol_D2_, respectively.

In summary, all hydrides investigated in this work have a comparable thermal stability, which is not significantly influenced by the relative amount of the transition metals. Indeed, the thermal behaviour is linked to the strength of the TM-D bond and correlates to the amount of energy necessary to break the bond to release D_2_ (H_2_). In complex hydrides, TM-D is a covalent bond, which means that the hydrides are stable, and relatively high temperatures are necessary for hydrogen desorption. In this case, the Fe-D and Co-D bonds have similar strengths and changing the amount of complex anions in the hydrides should not have a big influence on thermal stability and desorption temperatures.

### Structural and microstructural analysis after hydrogen desorption

The structural and microstructural analysis after hydrogen desorption was performed on samples after TPD analysis. The PXD patterns (Fig. S11) confirm previously reported results^4^. A bcc-(FeCo) solid solution and elemental Mg are observed, in samples from Fe0.3 to Fe0.9, while for the ternary Mg_2_FeD_6_ hydride, Fe and Mg form. An intermetallic Mg-Co compound is also observed in Co-rich samples, i.e. from Fe0.0 to Fe0.3. As mentioned previously, Mg and Co only form one stable intermetallic compound, MgCo_2_^9^. Other metastable phases, such as MgCo or Mg_2_Co, have been reported after the decomposition of Mg_2_CoH_5_^5,7,16,17,34^. The exact stoichiometry of the Mg-Co intermetallic compound formed after desorption of Mg_2_CoH_5_ is widely discussed in the literature^5,7,16,17,34^, since the temperature influences the nature of this product^17^, due to the formation of metastable intermetallic phases. Here it was not possible to define the exact stoichiometry or crystal structure of the observed Mg-Co compound, but we exclude that it is MgCo_2_ or MgCo, as no diffraction peaks match with the MgCo or MgCo_2_ structures.

Mg is hard to be detected in all samples (Fig. S11). This can be better visualized in Fig. S12, which shows the patterns after desorption for sample Fe0.3, Fe0.5 and Fe0.7. Quantitative analysis on the latter two results in Mg amounts less than 10 wt.% which are not representative of the nominal composition (≈ 46 wt.%).

**Figure 6:**
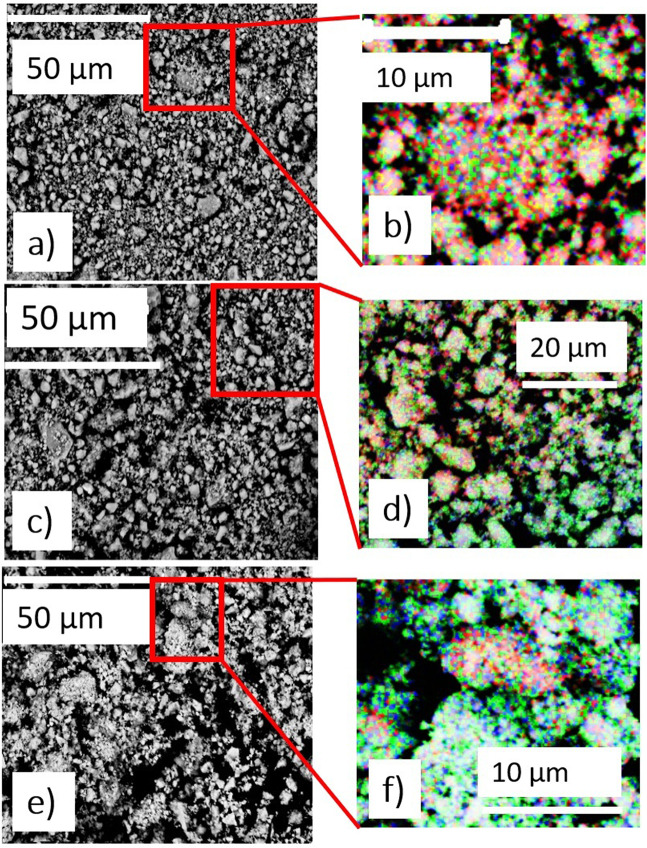
SEM micrographs of sample Fe0.7 (**a**) after milling (**c**) after annealing and (**e**) after desorption. EDS elemental mapping of the (**b**) as-milled, **(d**) annealed, and (**f**) desorbed samples are also shown with Mg (red), Fe (green) and Co (blu). The micrographs were all registered at magnification of X 5000. The scale is specified in each micrograph and given by the white bar.

To better understand the elemental distribution after desorption, a SEM analysis with EDS mapping was performed on sample Fe0.7, comparing powder morphologies and the distribution of Mg, Fe and Co after milling, after annealing and after decomposition. Reactive milling produces a fine powder morphology (Fig. 6a), and this is observed also in powders after annealing (Fig. 6c). The average particles size is of the order of 10 μm, with some powder agglomerations. In the desorbed powder (Fig. 6e), a similar powder morphology is present. The EDS maps for the as-milled powder (Fig. 6b), show a uniform distribution of Mg, Co and Fe elements, suggesting a homogeneous distribution of the hydride. Moreover, the measured weight percentage of elements, i.e. Mg 41 wt.%, Fe 40 wt.% and Co 19 wt.%, agrees well with the nominal composition, i.e. Mg 46 wt.%, Fe 37 wt.% and Co 17 wt.%, thus confirming the effectiveness of the milling process. The annealed sample (Fig. 6d), still presents a homogeneous distribution of the elements, representative of the quaternary hydride, but some regions with only Fe and Co are also observed. This indicates that annealing promotes the growth and phase segregation of the (FeCo) phase. The overall elemental composition (Mg 37 wt.%, Fe 43 wt.% and Co 20 wt.%) is very similar to that measured before annealing and agrees with the nominal composition. On the contrary, in the desorbed sample (Fig. 6f), Mg is confined to fewer regions and is detected in low quantities, whereas iron and cobalt are predominant. The result of the elemental analysis (Mg 5 wt.%, Fe 66 wt.% and Co 29 wt.%) agrees with what was found from PXD, suggesting that desorption causes a strong phases separation. Moreover, we cannot exclude a partial sublimation of Mg due to experimental condition of the TPD analysis (high vacuum and final temperature of 823 K).

### Estimation of the enthalpy of formation of the Mg-Fe-Co hydrides

The enthalpy of formation ΔH_f_ of the hydrides synthesized in this work can be estimated from the enthalpy of desorption ΔH_des_ measured by DSC, (see Table 1), and from literature and experimental values of ΔH_mix_ and ΔH_f_ of the desorbed products observed in PXD patterns (see Fig. S11). The value of ΔH_mix_ for FeCo is taken from ref. ^35^, while that for Mg-Co is estimated from the area of the exothermic peak observed in the DSC (see Fig. 5) and discussed in section 3.4. All the values used for the estimate are summarized in Table S1.

**Figure 7:**
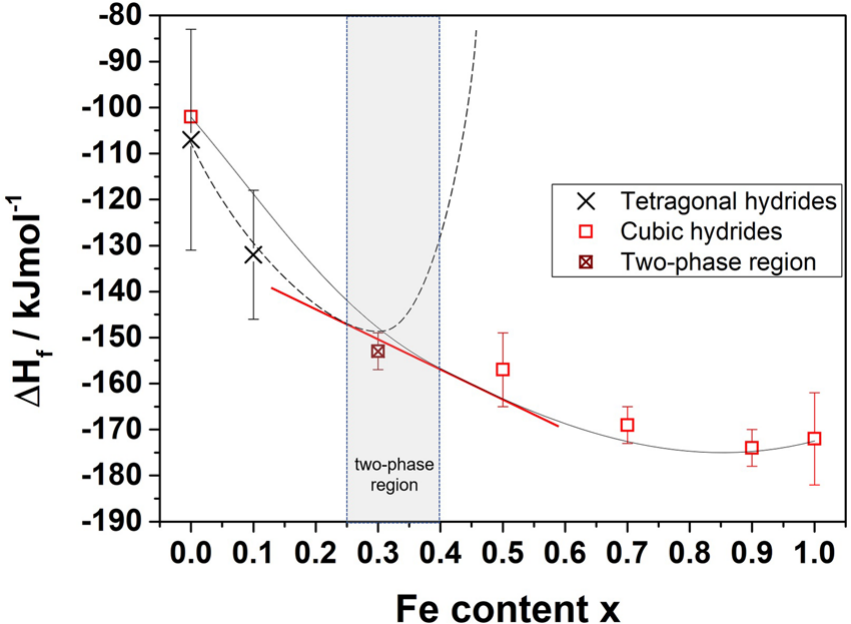
Estimated ΔH_f_ of the hydrides as a function of the relative Fe content (Mg_2_Fe_x_Co_1−x_D_y_).

The estimated values of ΔH_f_ can be plotted as a function of the nominal Fe content for assessing the stability of Mg-Fe-Co hydride phases with respect to the relative content of the TM (Fig. 7). The ΔH_f_ of the cubic phase changes from about −172 ± 10 kJ/mol_H2_ for Fe1.0 to −157 ± 8 kJ/mol_H2_ for Fe0.5, indicating a decreasing stability with increasing Co content. This trend can be schematically represented by the solid curve drawn in Fig. 7, where the estimated ΔH_f_ for the high temperature cubic phase of Mg_2_CoH_5_ is also taken into account. The latter was calculated by considering the enthalpy for the allotropic tetragonal-cubic transformation reported in ref. ^5^.

The trend for the stability of the tetragonal phase is schematically represented by the dashed line in Fig. 7. In this case, only two experimental points are available, one for Fe0.0 and one for Fe0.1, respectively. Nonetheless, the schematic curves takes into account the formation of a tetragonal phase reported for x = 0.25 by Zèlis *et al*.^23^, for which however neither ΔH_f_ nor ΔH_des_ were reported. For x > 0.25 we assume ΔH_f_ values increasing and extending up to positive values for higher Fe-contents, since no tetragonal phase has ever been reported for those compositions.

Based on the schematic representation in Fig. 7 and by applying the common tangent construction, we can identify a two-phase region for 0.25 < x < 0.4 where the tetragonal and cubic phases coexist. The occurrence of a phase mixture in such a region is supported by our results for Fe0.3, which show the presence of both tetragonal Mg_2_(FeH_6_)_0.3_(CoH_5_)_0.7_ and cubic Mg_2_(FeH_6_)_0.4_(CoH_5_)_0.6_. It should be stressed that the estimated ΔH_f_ for Fe0.3, which is listed in Table S1 and included in Fig. 7, is the value obtained from the desorption of both phases, since two separate endothermic peaks were not observed (see Fig. 5). Thus, an estimate of ΔH_f_ for each compound was not possible.

### Rehydrogenation of Mg_2_(FeH_6_)_0.5_(CoH_5_)_0.5_

In the literature, both Mg_2_FeH_6_^33^ and Mg_2_CoH_5_^5^ display reversible hydrogen sorption reactions, generally at high pressures (i.e. 80-100 bar H_2_) and temperatures (i.e. >673 K). However, for Mg_2_CoH_5_ reversibility has been demonstrated also at mild conditions, i.e 623 K and 20 bar of H_2_^30^, while the re-hydrogenation of Mg_2_FeH_6_ was also reported at 622 K and 5 bar of H_2_^36^.

The reversibility of hydrogen release and uptake reactions was tested at isothermal conditions at 673 K for sample Fe0.5 in the as-milled state. Desorption in vacuum was very fast and in less than 30 min all hydrogen was desorbed. On the contrary, a rather slow kinetics of absorption was observed at 10 bar of H_2_, with only 0.22 wt.% H_2_ absorbed in 35 hours. PXD measurements performed after absorption at 10 bar revealed that only a small amount of MgH_2_ were formed. Moreover, SEM-EDS analysis performed after absorption still show significant segregation of the unreacted FeCo and Mg phases, as observed for the samples after decomposition (section 3.5).

**Figure 8:**
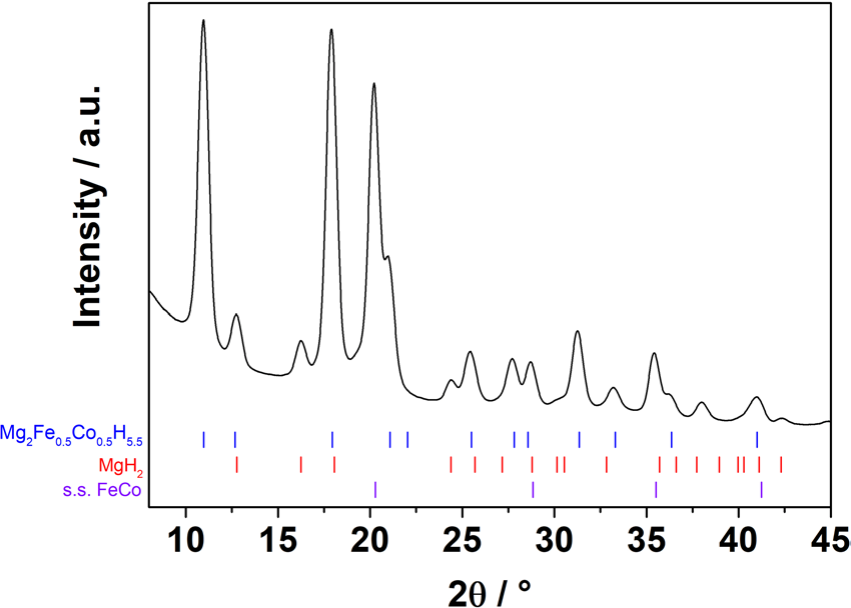
PXD pattern for sample Fe0.5 after rehydrogenation, which was performed at 30 bars of H_2_ and 673 K. The position of Bragg reflections for FeCo, Mg_2_Fe_0.5_Co_0.5_H_5.5_, MgH_2_ and FeCo is indicated by the bars at the bottom.

A much faster absorption kinetics is observed when the H_2_ pressure is increased up to 30 bar. Indeed, most of the hydrogen is absorbed within 2 hours and the final hydrogen content is 4.4 wt.% H_2_. This is comparable to the amount registered during desorption (i.e. 4.3 wt.% H_2_). PXD measurements on the sample after the rehydrogenation (Fig. 8) show that Mg_2_(FeH_6_)_0.5_(CoH_5_)_0.5_ is the main reaction product, while MgH_2_ is also formed as a secondary phase.

EDS elemental mapping show a quite uniform elemental distribution, as observed for the as-milled sample (section 3.5). The formation of the quaternary hydride Mg_2_(FeH_6_)_0.5_(CoH_5_)_0.5_ confirms that hydrogen sorption reactions are reversible also for the mixed transition metal quaternary systems. Rehydrogenation is possible thanks to the fine microstructure (Fig. 6e,f) and the high density of Mg/(FeCo) interfaces which are still present after desorption, allowing hydrogen to be reabsorbed. Mg_2_(FeH_6_)_0.5_(CoH_5_)_0.5_ has a fast kinetics of desorption at high temperature and rehydrogenation occurs with relatively fast kinetics at 30 bar.

## Conclusions

This work confirms the effectiveness of RBM for synthetizing Mg-based transition-metal complex hydrides from elemental powder at relatively low pressure and close to room temperature. Milling promotes mixing of the starting elemental powders and hydrogen absorption, the hydrides forming in less than 10 hours with high yields. The crystal structure characterization of the milling products shows that depending on the relative content of Fe and Co (Fe_x_Co_1−x_) the following hydrides form: Mg_2_(FeD_6_)_0.09_(CoD_5_)_0.91,_ Mg_2_(FeD_6_)_0.3_(CoD_5_)_0.7_, Mg_2_(FeD_6_)_0.4_(CoD_5_)_0.6_, Mg_2_(FeD_6_)_0.5_(CoD_5_)_0.5_, Mg_2_(FeD_6_)_0.7_(CoD_5_)_0.3_ and Mg_2_(FeD_6_)_0.9_(CoD_5_)_0.1_. Fe and Co randomly occupy the same crystallographic site, creating solid solutions containing both [FeD_6_]^4−^ and [CoD_5_]^4−^ complex anions. For x ≥ 5, the quaternary hydrides have the same cubic *Fm*$\bar{3}$*m* structure as Mg_2_FeD_6_ and Mg_2_CoD_5_ at high temperature, while for x < 0.3 the tetragonal structure *P*4/*nmm* of Mg_2_CoD_5_ at RT is observed. For x = 0.3, a two-phase region where the tetragonal and cubic phases coexist is found. The existence of the two-phase region is supported by the assessment of the stability of the hydrides from experimental and literature data. The activation energy Ea_des_ (87-89 kJ/mol) and enthalpy of desorption ΔH_des_ (about 70 kJ/mol) do not change significantly with the relative amount of complex anions. The quaternary hydrides synthesized in this work are quite stable, but their desorption temperature is below that of MgH_2_. Desorption of hydrogen results in the formation of Mg and (FeCo) solid solutions, which are found inhomogeneously distributed. Nonetheless, the reversible hydrogenation to form the quaternary hydrides is observed at 30 bar of H_2_ and 673 K for Mg_2_(FeH_6_)_0.5_(CoH_5_)_0.5_.

## Supplementary information

Supplementary Information.

## Data Availability

All datasets reported in this manuscript are available from the corresponding author on reasonable request.
