# Supplementary material for: Synthesis and characterization of Magnesium-Iron-Cobalt complex hydrides
Source: Sci Rep. 2020 Jun 2;10:9000. doi: 10.1038/s41598-020-65774-8 (PMC7265542; doi:10.1038/s41598-020-65774-8)
Supplement: Supplementary file 1 — Supplementary Information. [file 41598_2020_65774_MOESM1_ESM.pdf]

SUPPLEMENTARY INFORMATION

Synthesis and characterization of Magnesium-Iron-Cobalt complex hydrides

Jussara Barale,[a)b)] Stefano Deledda,[b)] Erika M. Dematteis,[a)] Magnus H. Sørby,[b)] Marcello Baricco[a)] and Bjørn C. Hauback[b)]

[a)] Department of Chemistry, Inter-departmental Center Nanostructured Interfaces and Surfaces (NIS), and INSTM, University of Turin, Via Pietro Giuria 7, 10125 Torino, Italy

[b)] Department for Neutron Materials Characterization, Institute for Energy Technology (IFE), PO Box 40, NO-2027 Kjeller, Norway

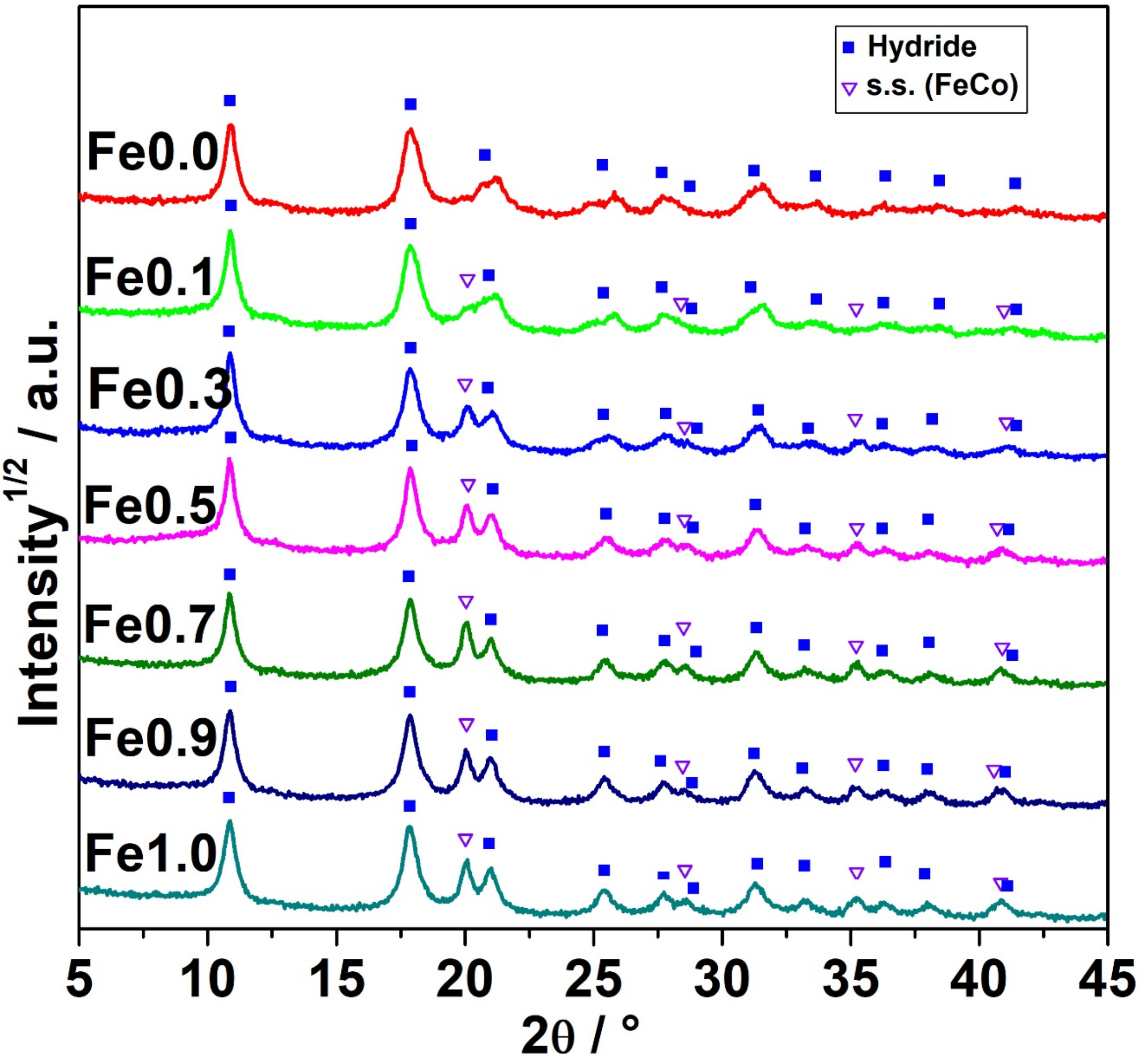

Figure S1 – Powder X-ray Diffraction patterns of all samples annealed at 473 K in 50 bars of D_2 for 48 hours. Full blue squares refer to hydride phase, while empty violet triangles to the solid solution (FeCo).

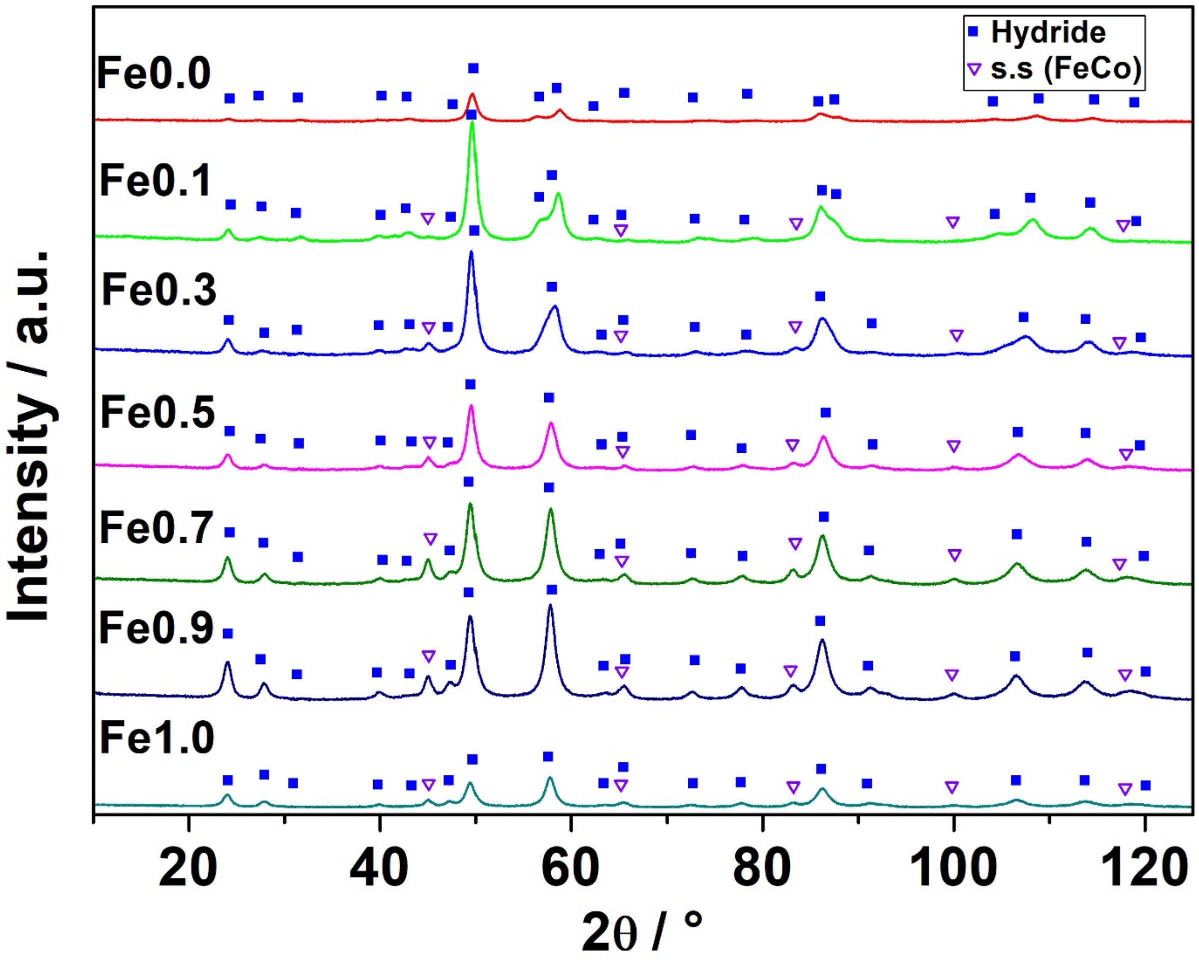

Figure S2 – Powder Neutron Diffraction patterns of all samples annealed at 473 K in 50 bars of D_2 for 48 hours. Full blue squares refer to hydride phase, while empty violet triangles to the solid solution (FeCo).

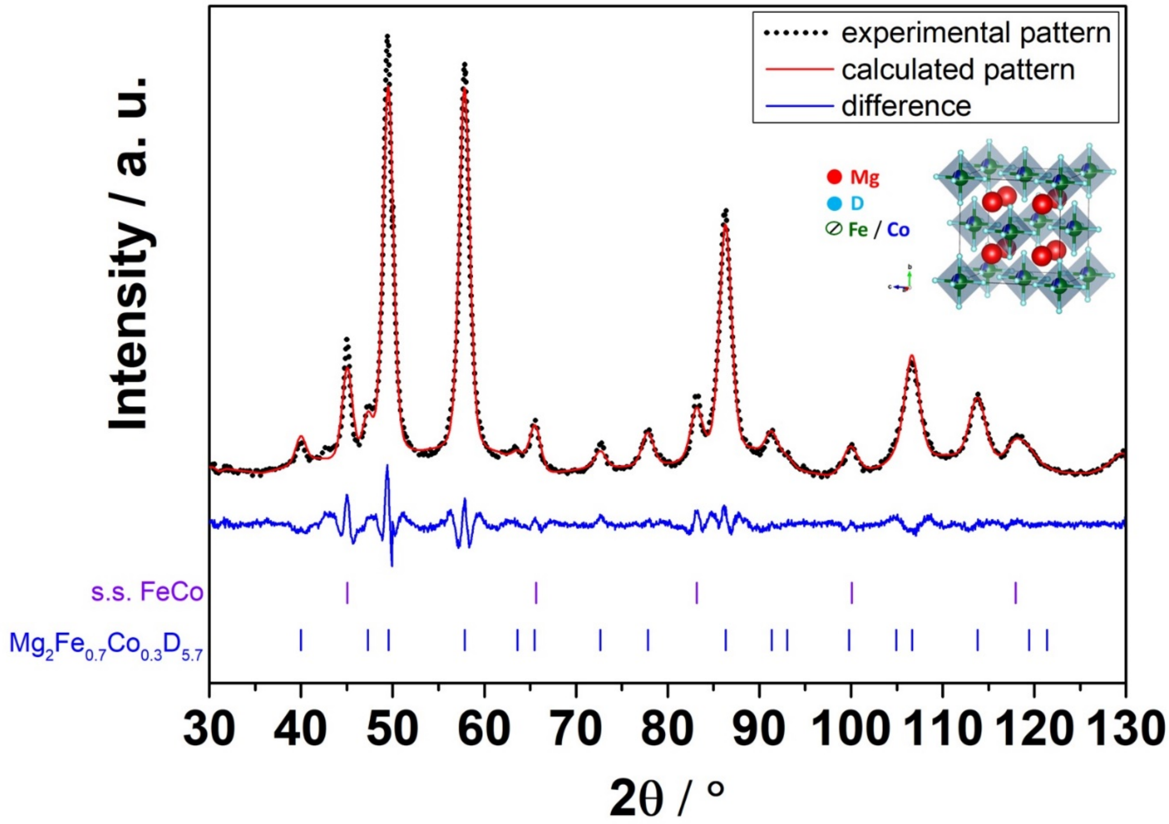

Figure S3 – Experimental (black dots) and calculated (red line) Powder Neutron Diffraction pattern of sample Fe0.7 after annealing. The blue line shows the difference between observed and calculated intensities together with the calculated crystal structure. Peaks assignation is reported. A sketch of the crystal structure is also reported.

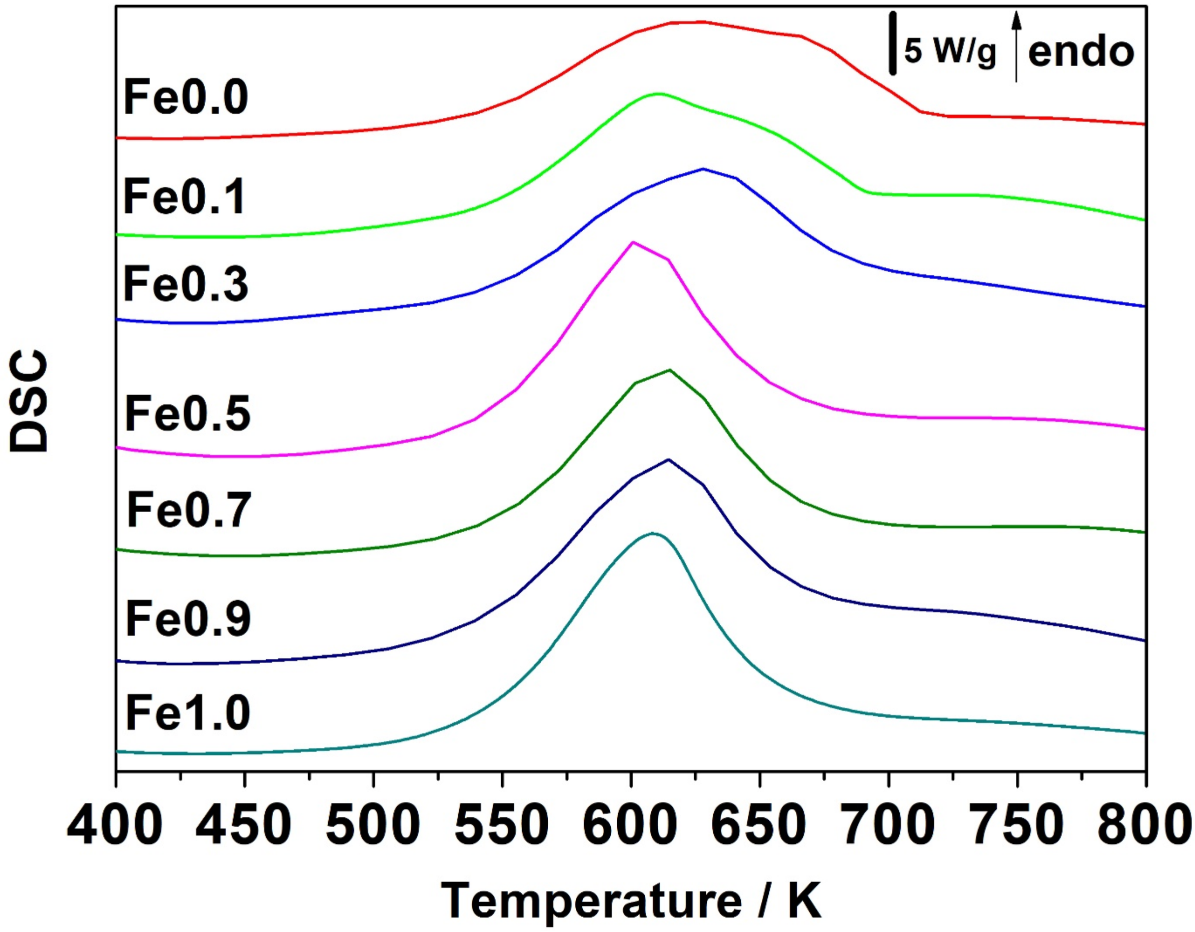

Figure S4 - DSC traces as a function of temperature recorded at 40 K/min.

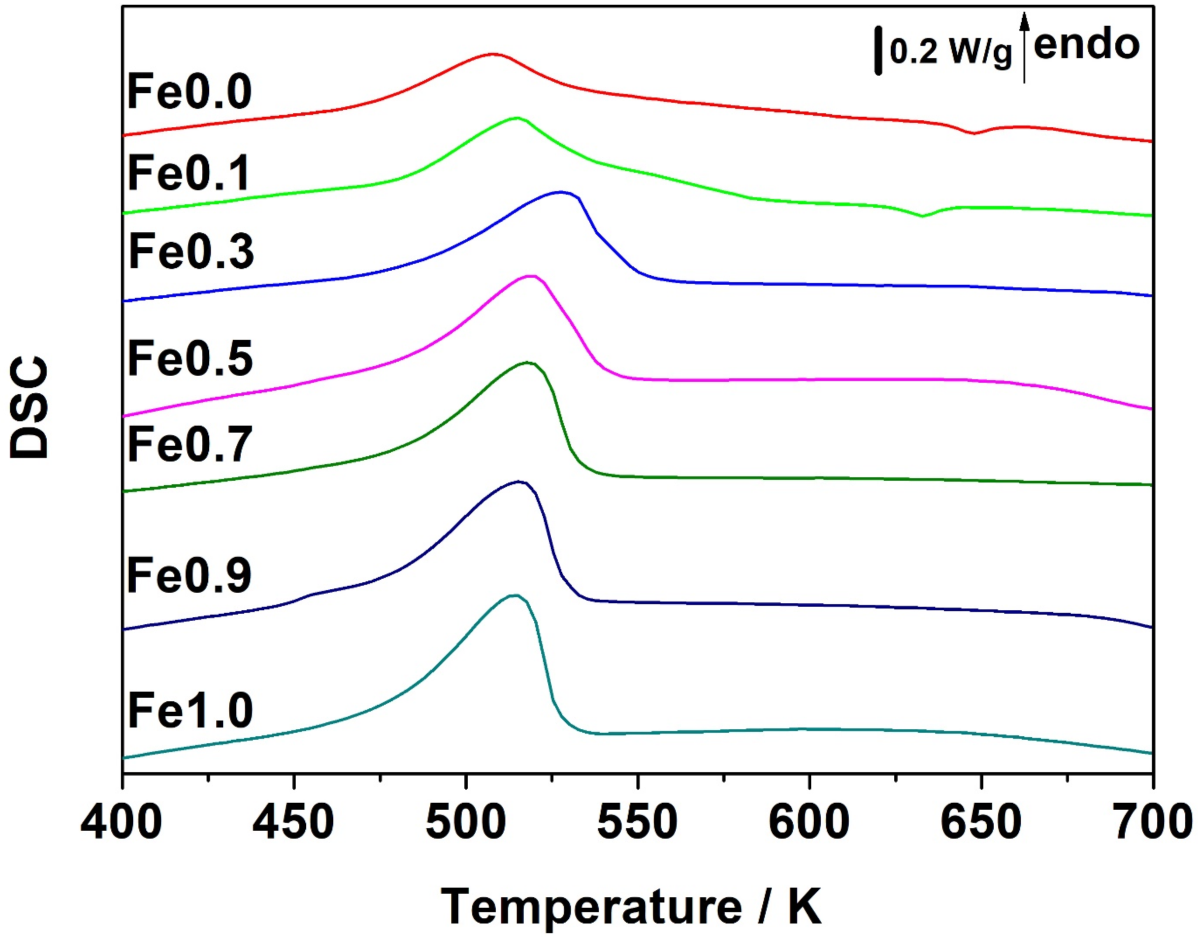

Figure S5 - DSC traces as a function of temperature recorded at 1 K/min.

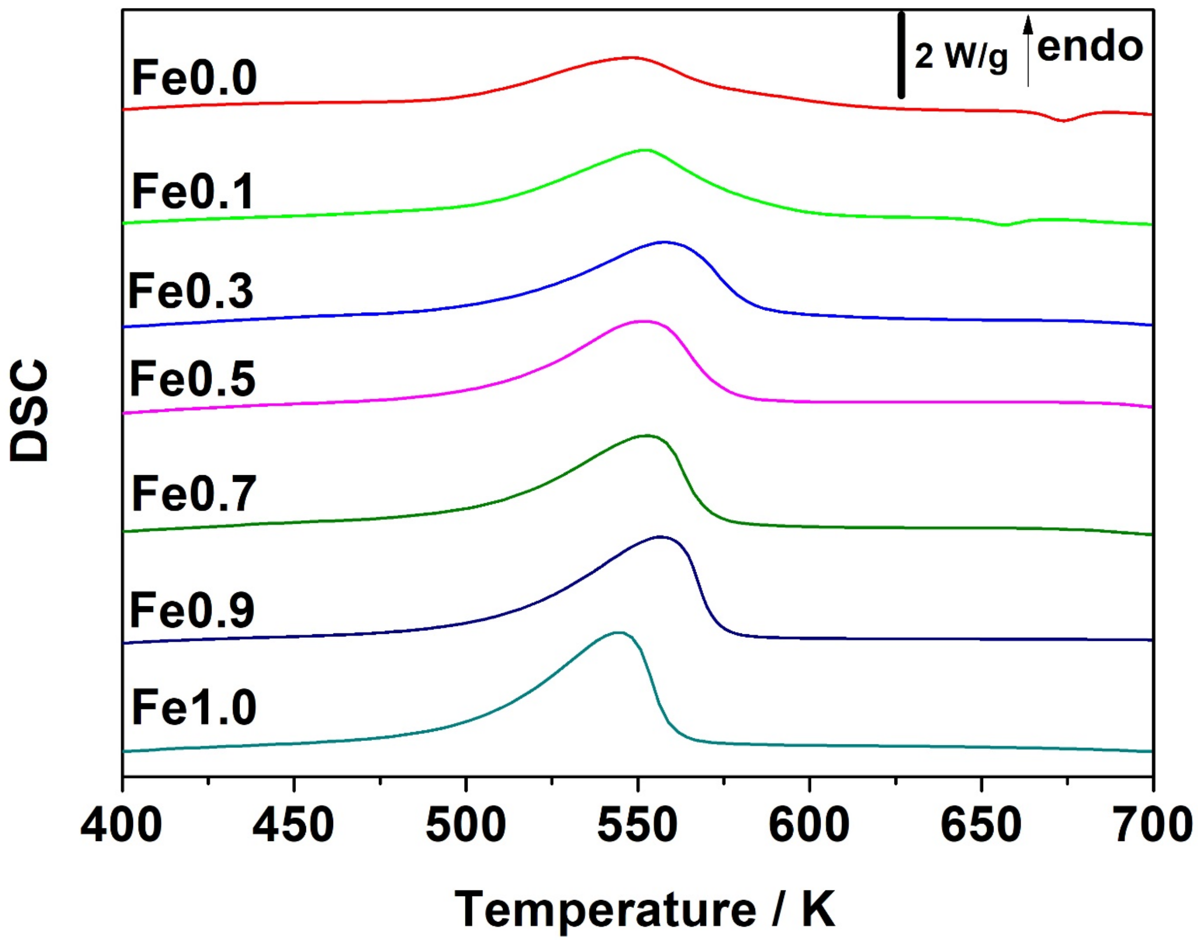

Figure S6 – DSC traces obtained at 5 K/min for all samples.

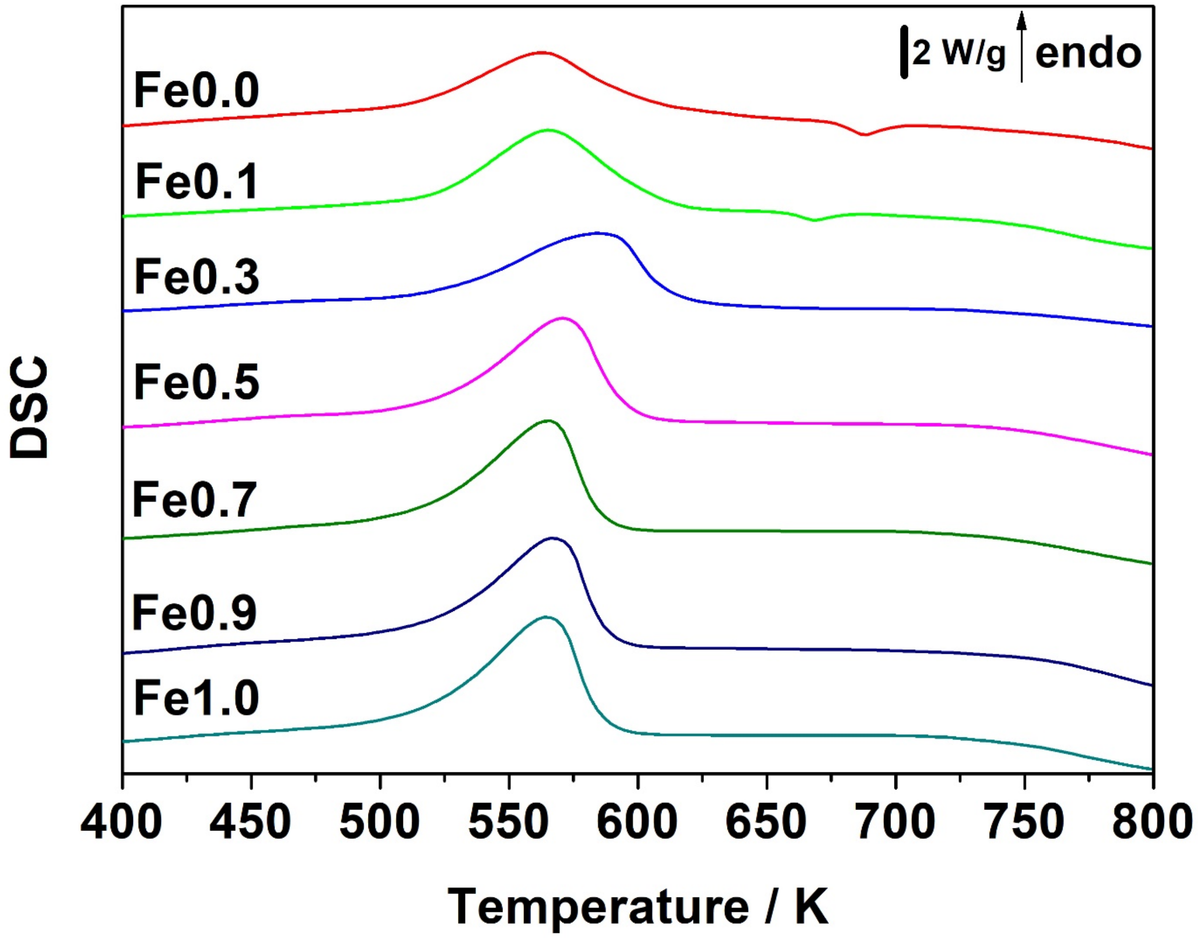

Figure S7 - DSC traces as a function of temperature recorded at 10 K/min.

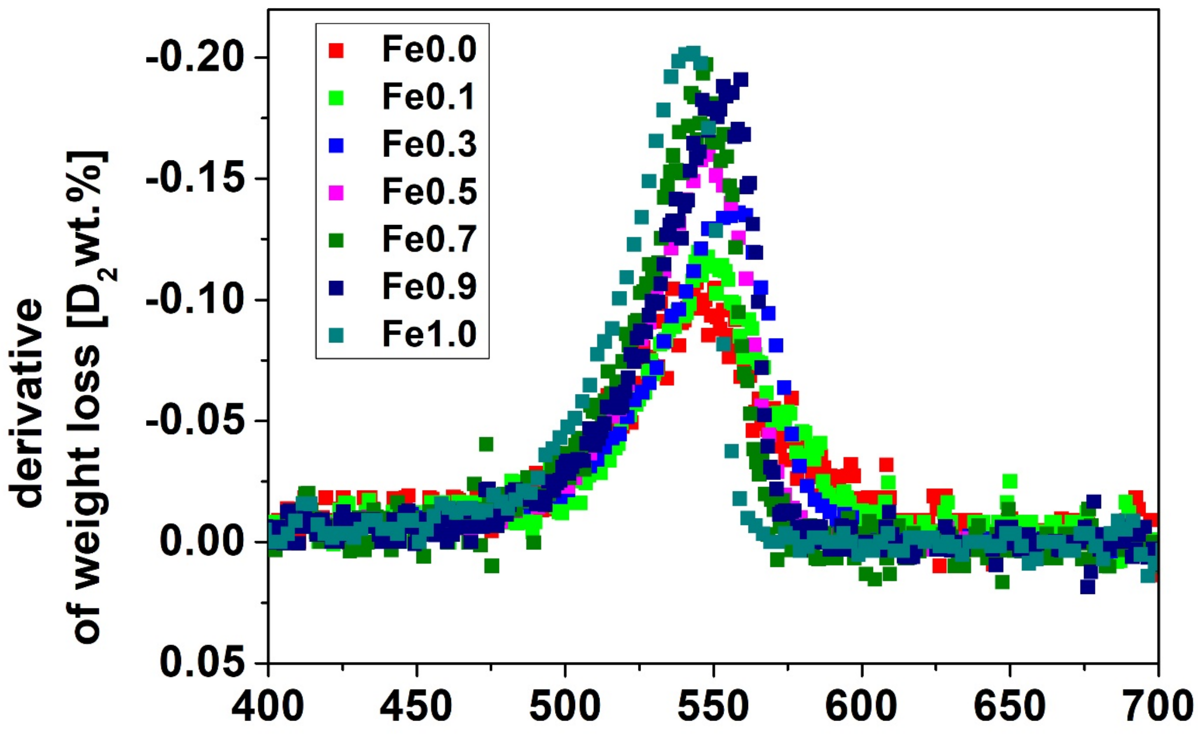

Figure S8 –Derivate of TG curves obtained at 5 K/min for all samples.

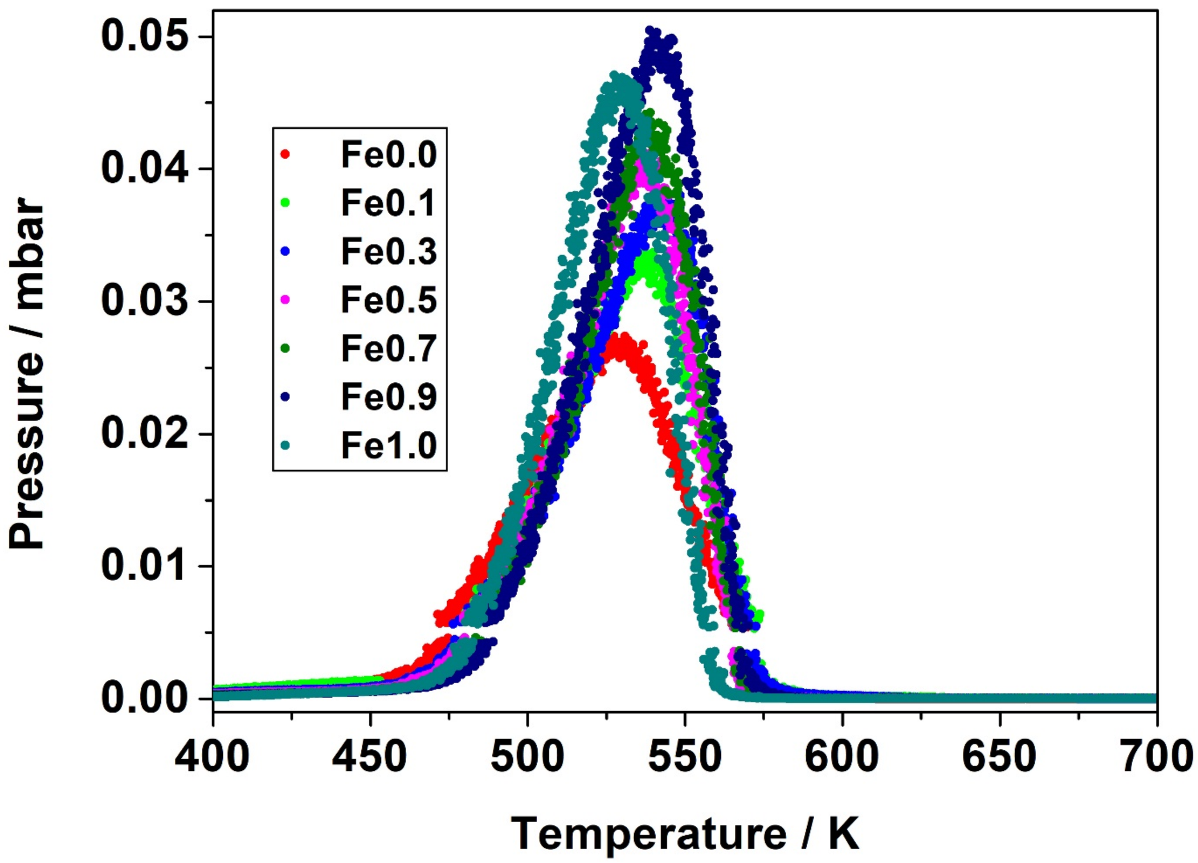

Figure S9 – TPD signals obtained at 5 K/min for all samples. Two different pressure sensors are used during measurement, resulting in an interruption of the signal when switching from one to the other.

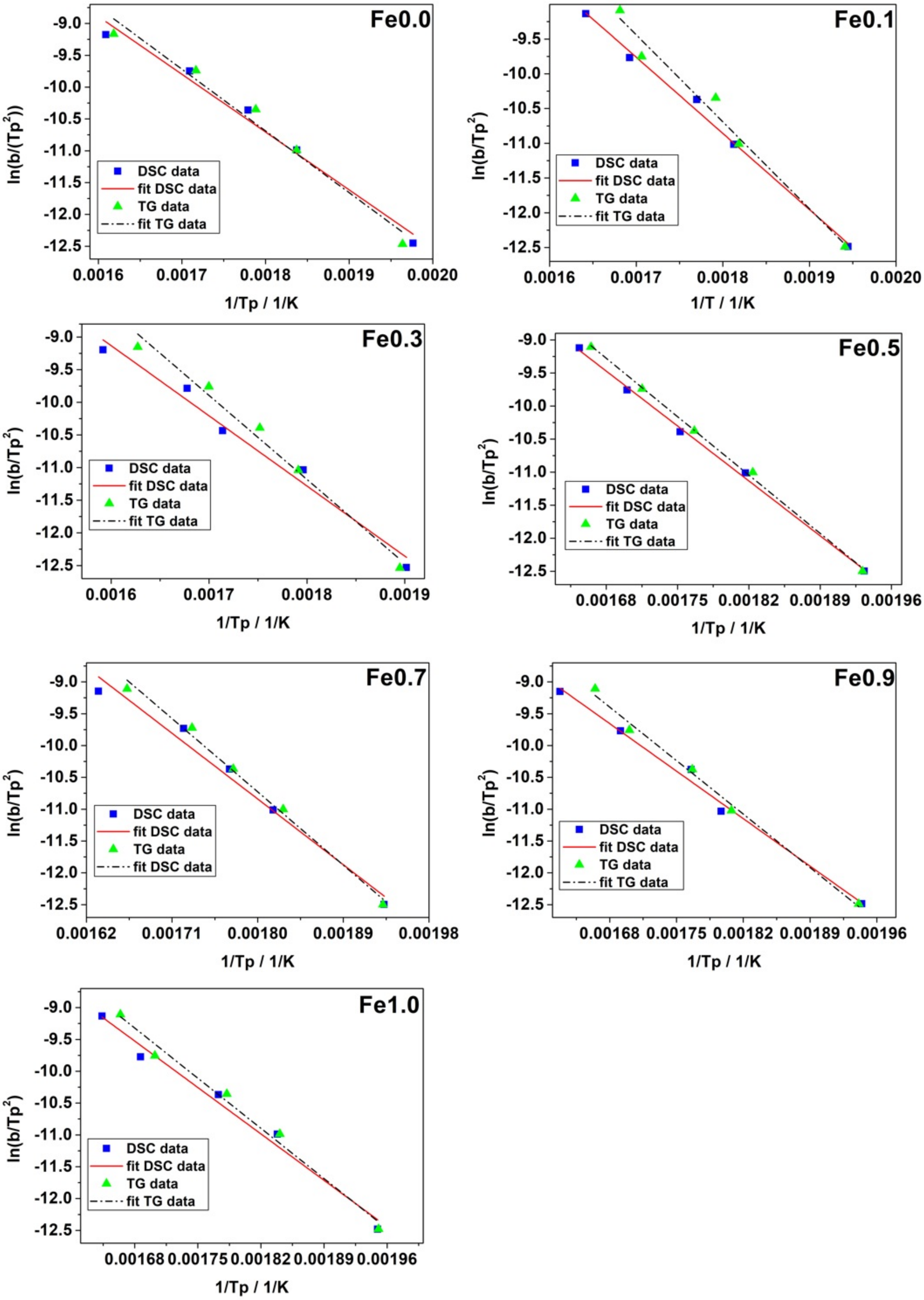

Figure S10 - Kissinger Plots obtained from DSC and TG curves for all samples. The peak temperature Tp corresponds to the maximum DSC peak temperature or the inflection point of the TG curve.

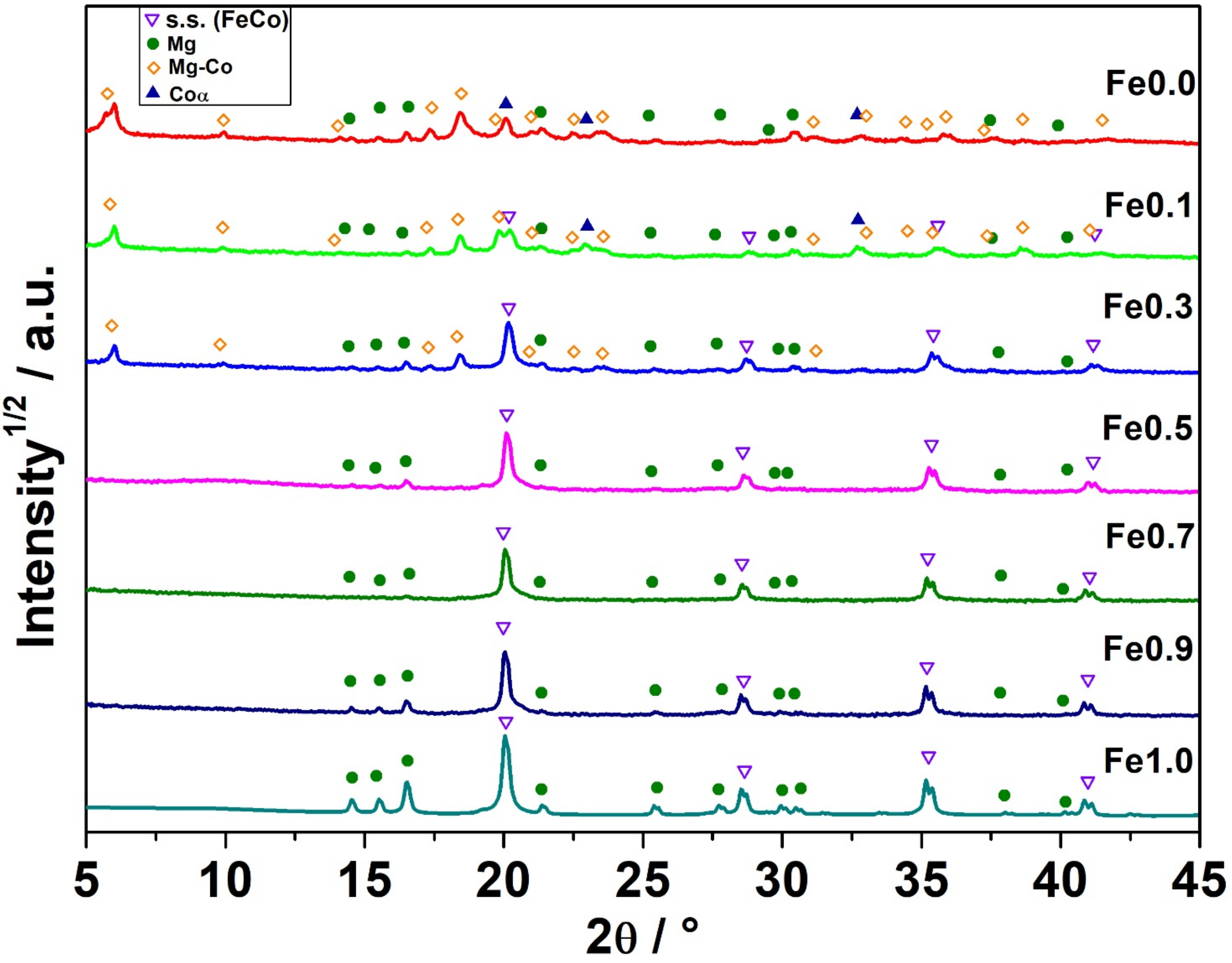

Figure S11 – Powder X-ray Diffraction patterns of samples after desorption obtained from TPD measure. Full green dots refer to Mg phase, full blue triangles to Co-α, empty violet triangles to the solid solution (FeCo) and empty orange rhombuses to the intermetallic compound Mg-Co.

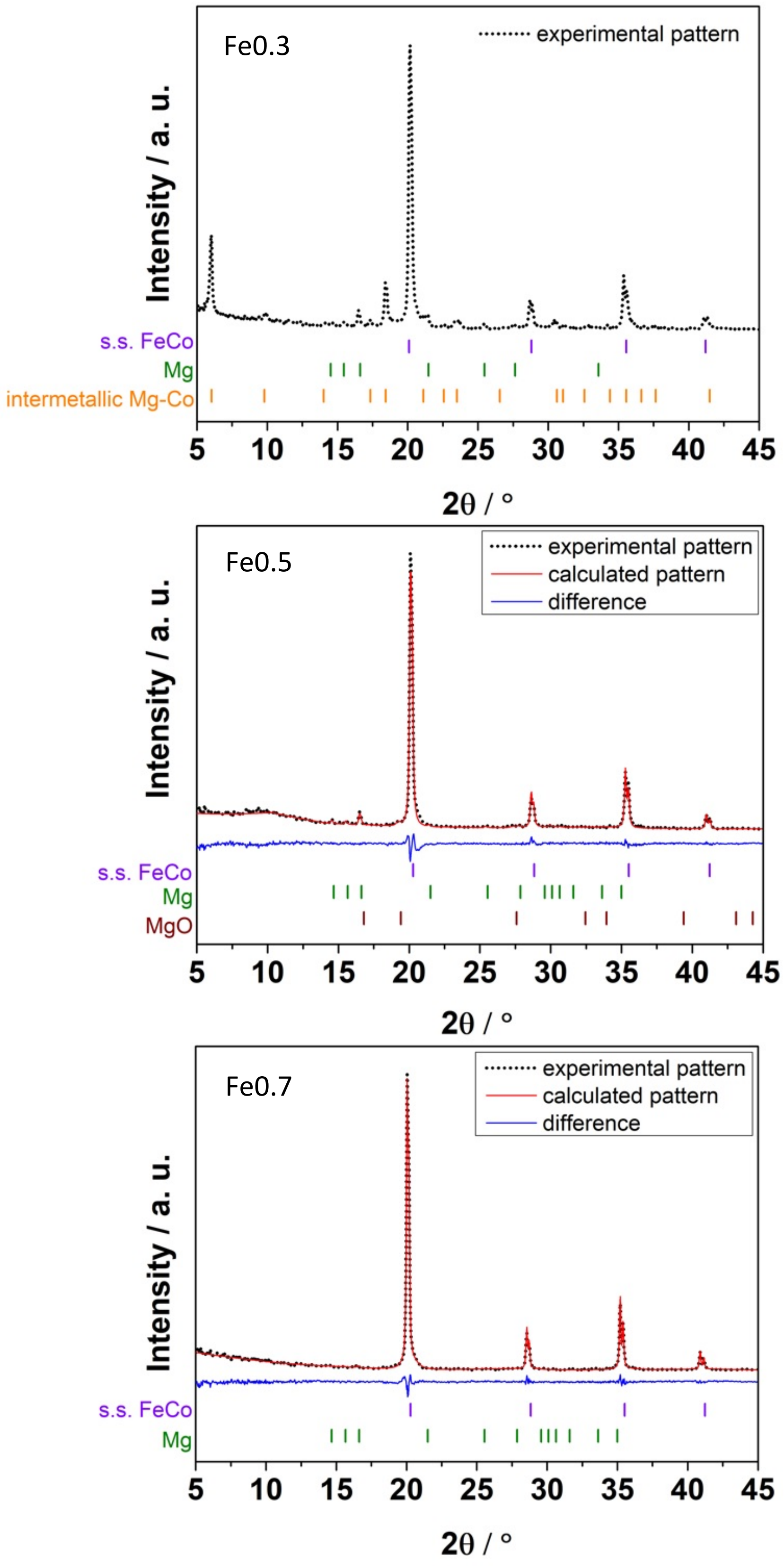

Figure S12 – PXD pattern of Fe0.3, Fe0.5 and Fe0.7, obtained from TPD analysis, with Rietveld refinement of the patterns of the last two samples. The experimental pattern is shown with black dots, while the calculated intensities in red line and their difference in blue line. Peak assignation is also reported.

TABLE S1 – For each sample, the symmetry, the estimated ΔH_f and the enthalpy values used to calculate it are summarized.

Sample	Symmetry	Estimated ΔH_f $kJmol^{-1}$	ΔH values used of desorbed products $kJmol^{-1}$
**Fe0.0**	P4/nmm	-107 ± 24	ΔH_{des}(hydride) = 104±24[a] ; ΔH_{mix}(Mg-Co) = 3[a]
	$Fm\bar{3}m$	102	ΔH_{des}(hydride) = 104±24[a] ; ΔH_{mix}(allotropic) ≈ 5[b] ; ΔH_{mix}(Mg-Co) = 3[a]
**Fe0.1**	P4/nmm	-132 ± 14	ΔH_{des}(hydride) = 131±4[a] ; ΔH_{mix}(FeCo) = 10[c] ; ΔH_{mix}(Mg-Co) = 2[a]
**Fe0.3**	P4/nmm	-153 ± 4	ΔH_{des}(hydride) = 142±4[a] ; ΔH_{mix}(FeCo) = 10[c] ; ΔH_{mix}(Mg-Co) = 1[a]
	$Fm\bar{3}m$		
**Fe0.5**	$Fm\bar{3}m$	-157 ± 8	ΔH_{des}(hydride) = 147±8[a] ; ΔH_{mix}(FeCo) = 10[c]
**Fe0.7**	$Fm\bar{3}m$	-169 ± 4	ΔH_{des}(hydride) = 159±4[a] ; ΔH_{mix}(FeCo) = 10[c]
**Fe0.9**	$Fm\bar{3}m$	-174 ± 4	ΔH_{des}(hydride) = 164±4[a] ; ΔH_{mix}(FeCo) = 10[c]
**Fe1.0**	$Fm\bar{3}m$	-172 ± 10	ΔH_{des}(hydride) = 172± 10[a]

a: experimental value from this work
b: ref. [5] M. Norek, T.K. Nielsen, M. Polanski, I. Kunce, T. Płociński, L.R. Jaroszewicz, Y. Cerenius, T.R. Jensen, J. Bystrzycki, Synthesis and decomposition mechanisms of ternary Mg2CoH 5 studied using in situ synchrotron X-ray diffraction, Int. J. Hydrogen Energy. 36 (2011) 10760–10770.
c: ref. [35] I. Ohnuma, H. Enoki, O. Ikeda, R. Kainuma, Phase equilibria in the Fe – Co binary system, Acta Mater. 50 (2002) 379–393
